# *In Silico* Approach in Designing a Novel Multi-Epitope Vaccine Candidate against Non-Small Cell Lung Cancer with Overexpressed G Protein-Coupled Receptor 56

**DOI:** 10.31557/APJCP.2020.21.8.2297

**Published:** 2020-08

**Authors:** Leana Rich M Herrera

**Affiliations:** *Department of Physical Sciences, College of Science, Polytechnic University of the Philippines, Manila City, Philippines. *

**Keywords:** NSCLC, GRP56, immunoinformatics, vaccine

## Abstract

**Background:**

Majority of cancer-related deaths worldwide is attributed to non-small cell lung cancer (NSCLC). G protein-coupled receptor 56 (GPR56) is overexpressed and associated in the progression of NSCLC. The aim of this study is to use immunoinformatics approach in designing a multi-epitope vaccine to target overexpressed GPR56 which can potentially activate antibody-mediated cell death mechanisms and inhibit pathways involved in the proliferation, migration and survival of NSCLC.

**Methods:**

Herein, the reported overexpression of GPR56 was further investigated by conducting a differential gene expression analysis of NSCLC samples from GEO DataSets (GSE29249). Results confirmed significant overexpression of GPR56 in NSCLC compared to adjacent normal samples. A multi-epitope vaccine (Fvax) was constructed in silico by adjoining B lymphocytes (BL) and helper T lymphocytes (HTL) epitopes from the extracellular sequence of GPR56. Population coverage (PC) of HTL epitopes was also estimated. To enhance its immunogenicity, sequences of flagellin domains were fused as adjuvant. Fvax was evaluated in silico for antigenicity, allergenicity, peptide toxicity, physicochemical properties and cross-reactivity. Its tertiary structure was predicted, refined, and validated followed by structural epitope prediction. Lastly, Fvax DNA was optimized and cloned in silico.

**Results:**

This is the first work to design a potential vaccine against GPR56-overexpressing NSCLC. Fvax has 3 BL and 7 HTL immunogenic epitopes on GPR56. In silico evaluations suggest that Fvax is antigenic, non-toxic, non-allergenic, stable, and has accessible BL epitopes with high PC worldwide for HTL epitopes.

**Conclusion:**

Overall, results showed that Fvax is a potential vaccine against NSCLC. The approach of this study efficiently minimized the number of tests, cost and time required to select the best epitopes and to design a vaccine for the treatment of NSCLC.

## Introduction

Lung cancer is the major cause of cancer-related deaths globally (Bray et al., 2018). Approximately 87% of lung cancer cases are non-small cell lung cancer (NSCLC) (Grapatsas et al., 2017). Patients with NSCLC often have different therapeutic needs depending on the tumor presentation. Combinations of chemotherapeutic agents, radiation therapy, immunotherapy, and surgery are currently used; however, these are often accompanied by tumor regression, metastasis and adverse events. To date, there is no single remedy available to completely treat NSCLC due to its complications. 

Advancements in the understanding of the immune system lead to the development of immunotherapy against cancer. Studies on cancer vaccines targeting tumor-associated antigens (TAA) have recently entered clinical trials with encouraging results (Kotsakis et al., 2014; Mizukoshi et al., 2015). TAA includes overexpressed signalling receptors on the surface of cancer cells. G protein-coupled receptors (GPRs) are signalling receptors playing important roles in cell signal transductions, cell adhesion and cell-cell interactions. GPRs have been implicated in the development of diseases like cancer, one of these is the G Protein-Coupled Receptor 56 (GPR56) with 693 amino acid (aa) residues, encoded by Adhesion G-Protein Coupled Receptor G1 (ADGRG1) gene. Immunohistochemistry and overexpression studies showed that GPR56 is overexpressed in NSCLC and is involved in promoting its proliferation and invasion (Ke et al., 2007; Song et al., 2016). Moreover, Song and colleagues (2016) found that the expression of GPR56 is correlated with the TNM stage of NSCLC. Overexpression of GPR56 has been also associated in the progression of other cancer types (Shashidhar et al., 2005; Zhang et al., 2019). Taking advantage of the overexpression and roles of GPR56 in NSCLC, designing a vaccine containing BL and HTL epitopes to induce immune responses against GPR56-overexpressing NSCLC is a potent strategy.

One of the requirements to activate TAA-specific helper T lymphocytes (HTL) which has escaped clonal deletion is to break the natural immune tolerance towards that TAA. A signal must be triggered by pathogen-derived molecules containing pathogen-associated molecular patterns (PAMPs) which are recognized by pathogen-recognition receptors (PRR) expressed on the surface of immune and non-immune cells. Among these PRRs are toll-like receptors (TLR). TLR5 is a member of TLR family known to bind and recognize flagellin (Hayashi et al., 2001). Flagellin is a pathogen-associated protein found in flagellated bacteria, and has been successfully employed as safe and strong adjuvant to vaccines in clinical trials (Treanor et al., 2010; Taylor et al., 2011). Thus, this work incorporated flagellin domains from Salmonella typhimurium (fliC) as adjuvant.

The main purpose of this work is to design a novel multi-epitope vaccine against GPR56-overexpressing NSCLC, which can also potentially augment available therapies. Peptide-based vaccines can elicit immune responses against cancer cells (Saiag et al., 2016). The development of peptide-based vaccines has been more efficient and cost-effective through the use of in silico techniques and bioinformatics tools. 

## Materials and Methods

*Confirmation of the overexpression of GPR56 in NSCLC*

Prior to this study, the overexpression of GPR56 in NSCLC and various cancer types have been reported. As a confirmatory step, gene expression profile of ADGRG1 (encoding GRP56) from Gene Expression Omnibus (GEO) database (GSE29249) was analyzed with GEO2R. Tissue profiles from NSCLC samples (n=6) were compared to normal adjacent samples (n=6). Data log transformation was applied in the analysis.

*Prediction of candidate linear B lymphocyte epitopes*

The extracellular sequence of GPR56 (Q9Y653) with 377 aa (26-402) was retrieved from UniProt database to predict linear BL epitopes using multiple tools. Default thresholds in Emini Surface Accessibility (ESA), BepiPred Linear Epitope (BLE), and Kolaskar and Tongaonkar Antigenicity (KTA) tools at Immune Epitope Database (IEDB) and ABCPred Server were used to generate linear BL epitopes. Consensus BL epitopes with 20 residues from at least 2 servers were further assessed for antigenicity, allergenicity, and toxicity. 

*Prediction of helper T lymphocyte epitopes*

The binding affinity of HTL epitopes were predicted from the extracellular sequence of GPR56 using SMM-align method in NetMHCII 1.1 of IEDB Server. This method was validated to be superior to other MHC II epitope prediction methods (Nielsen et al., 2007). HTL epitopes were retrieved using the most frequent MHC II alleles (Greenbaum et al., 2011), and IC_50_ <500 nM which classifies epitopes as good binders (Jensen et al., 2018). Consensus sequences binding to at least 4 of the most frequent MHC II alleles with 15 residues were further evaluated for antigenicity, allergenicity and toxicity.

*Evaluation of antigenicity, allergenicity and toxicity of epitopes*

Antigenic epitopes were identified in Vaxijen 2.0 server using tumor as target with threshold ≥ 0.5. Vaxijen is an alignment-independent method which predicts antigen with 70%-89% accuracy (Doytchinova and Flower, 2007). Potential allergenic sites were assessed using AllergenFP v 1.0. The highest Tanimoto similarity index of an epitope matching an allergen from the database is used to identify whether the epitope is a probable allergen (Dimitrov et al., 2014). Lastly, to avoid potential toxicity, epitopes were scanned in ToxinPred server using default SVM parameters. 

*Estimation of population coverage*

The use of multi-epitopes could have larger population coverage (PC). In this study, the set of candidate HTL epitopes with their corresponding MHC II alleles was queried to estimate the worldwide PC using the Population Coverage tool in IEDB. In addition, PC was also calculated in areas with higher incidence rate of lung cancer including USA, Japan, China and countries that are in Europe (Barta et al., 2019). 

*Construction of Fvax*

Candidate BL and HTL epitopes were adjoined together using GPGPG linkers. Amino acid sequences of fliC (P06179) were linked as an adjuvant. FliC N-terminal region from D0-D1 (2-175) was adjoined with the N-terminus of multi-epitope construct via flexible EAAAK linker. Then, the C-terminus of the construct was fused with the C-terminal region of fliC D1-D0 (398-495) via EAAAK linker. Valine was added at the N-terminus of the construct to increase its half-life. To aid in efficient purification, 6-histidine tag was added at the C-terminus.

*Evaluation of cross-reactivity, antigenicity, allergenicity, and toxicity*

Possible cross-reactivity of Fvax against proteins expressed in Homo sapiens was evaluated using protein-protein BLAST (BLASTp). Default parameters were used to search for the possible hits against UniProtKB and SwissProt databases. Allergenicity, antigenicity and toxicity of the whole construct were further assessed in AllergenFP v.1.0, Vaxijen 2.0, and ToxinPred server, respectively. 

*Estimation of physicochemical properties of Fvax*

Molecular weight, amino acid composition, isoelectric point (pI), half-life, instability index, thermostability (aliphatic index) and the grand average hydropathicity (GRAVY) of Fvax were calculated using ProtParam tool in ExPASy (https://web.expasy.org/protparam/).

*Secondary and tertiary structure prediction, refinement and validation of Fvax*

Percentage composition of the secondary structures in Fvax was estimated using GOR4 tool (https://npsa-prabi.ibcp.fr/NPSA/npsa_gor4.html). RaptorX was used to identify solvent accessible and disordered regions (http://raptorx.uchicago.edu/StructurePropertyPred/predict/) in the vaccine. The tertiary structure of Fvax was predicted using GalaxyTBM tool (http://galaxy.seoklab.org/cgi-bin/submit.cgi?type=TBM) which employs multiple-template approach and was evaluated to be one of the top TBM servers (Ko et al., 2012). The initial structures were refined using GalaxyRefine tool (http://galaxy.seoklab.org/cgi-bin/submit.cgi?type=REFINE). To check the validity and to compare the initial and refined structures, ERRAT, ProSA-web, and PROCHECK tools were employed. ERRAT (https://servicesn.mbi.ucla.edu/ERRAT/) analyzes nonbonded atom-to-atom interactions in the input structure as compared to x-ray crystallography structures (Colovos and Yeates, 1993). ProSa-web server (https://prosa.services.came.sbg.ac.at/prosa.php) calculates the z-score which estimates the deviation of the structure from the validated x-ray crystallography and NMR structures of native proteins (Wiederstein and Sippl, 2007). PROCHECK (https://servicesn.mbi.ucla.edu/PROCHECK/) gives Ramachandran plots and shows the percentage of residues lying within the most favoured, and disallowed regions. A model with good quality is expected to have at least 90% of residues within the most favoured region (Laskowski et al., 1993). The best tertiary structure model for Fvax was viewed using PyMol and was further assessed for structural BL epitopes.

*Prediction of structure-based epitopes on Fvax*

The sequence of epitopes must be accessible and protruded enough so BCRs can bind to it. Ellipro (http://tools.iedb.org/ellipro/) predicts discontinuous and linear BL epitopes based from the protrusion index (PI) of a residue, and provides PI score for each protruded sequence. This tool was used to identify structural epitopes in Fvax.

*In silico cloning optimization of Fvax*

For the efficient cloning of Fvax in E. coli K12 strain, in silico codon optimization was conducted using Java Codon Adaptation Tool (JCAT) (http://www.jcat.de/). It calculates the GC-content and the Codon Adaptation Index (CAI) used to approximate the level of gene expression. The closer the CAI-value to 1, the more it is expressed in a host (Grote et al., 2005). Finally, the optimized sequence of Fvax was inserted into pET-30(+) as a vector using SnapGene tool.

## Results

*Overexpression of GPR56 in NSCLC tissue samples*

**Figure1:**
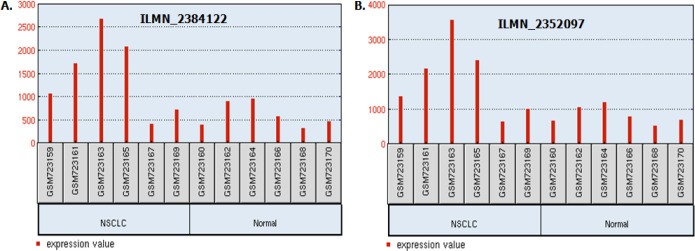
Expression values of ADGRG1 in NSCLC and adjacent normal tissue samples. Probe ILMN_2384122 (A); and ILMN_2352097 (B). Both probes show overexpression of ADGRG1 in NSCLC relative to normal adjacent tissue samples

Results showed that ADGRG1 is overexpressed approximately twice higher in NSCLC than the normal adjacent tissues. Probe ILMN_2384122 shows significant differential gene expression between NSCLC group and normal group with p-value 0.040, and 1.1 log2-fold change NSCLC/normal or 2.144–fold increase (Figure 1A). While probe ILMN_2352097 has p-value 0.036 and 0.992 log2-fold change NSCLC/normal or 1.99–fold increase (Figure 1B) conceding with the result of the first probe.

*Candidate linear BL epitopes*

ESA, BLE, KTA, and ABCPred prediction tools resulted to initial linear BL epitopes with 1-76 residues. From these epitopes, 3 epitopes (20aa) consensus from at least 2 servers, are classified to be antigenic, non-allergenic and non-toxic. Table 1 shows the sequences of candidate BL epitopes and their corresponding antigenicity scores, start, and end positions in GPR56.

*Candidate HTL epitopes*

Resulting epitopes from NetMHCII 1.1 were trimmed down to 7 HTL epitopes (15aa). Each has a good binding affinity to at least 4 of the most common human MHC II alleles and are classified as antigenic, non-allergenic, and non-toxic. Table 2 shows the sequences of the 7 candidate HTL epitopes and their corresponding antigenicity scores, MHC II alleles, minimum to maximum IC50 values, start, and end positions in GPR56.

*Population coverage of HTL epitopes*

The estimated worldwide PC for the set of HTL epitopes in Fvax is 83.81%. Moreover, the PC for the epitopes in areas with high incidence rates of lung cancer are 87.47% in Europe, 60.8 % in China, 75.04% in Japan, and 90.2% in USA.

*Fvax construct*

**Figure 2:**
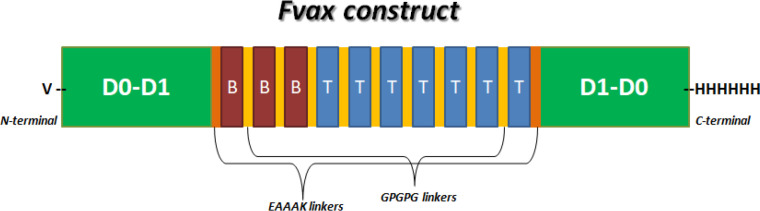
Schematic Representation of Fvax Construct

Figure 2 shows a schematic presentation of the whole Fvax construct. The N-terminus has a valine residue while the C-terminus has 6-histidine-tag. Candidate BL epitopes 113-132, 157-176, 317-336 (red) and HTL epitopes 204-218, 347-361, 313-327, 187-201, 88-102, 156-170, 204-218 (blue) are adjoined via GPGPG linkers (yellow). The D0-D1 N-terminal and C-terminal regions (green) from fliC are linked to the multi-epitope construct using EAAAK linkers (orange). 

*Cross-reactivity, antigenicity and toxicity of Fvax*

Fvax has no significant sequence similarity with other known human proteins except for GPR56 which implies specificity towards the target. The percentage identity of Fvax with GPR56 is 54.13% (E-value 2e-20) indicating significant sequence similarity. Fvax is classified as antigenic (0.6306) in tumor target, and is also non-allergenic with highest Tanimoto similarity index (0.87) to Q9Y4F5. Furthermore, no toxic peptide sequences were identified within the whole construct.

*Physicochemical properties of Fvax*

Fvax is consist of 499 residues with molecular weight of 52,707.25 Da, and pI of 6.37 which can be used for its purification using isoelectric focusing. The estimated half-life of Fvax is 100 hours in mammalian reticulocytes in vitro, >20 hours in yeast in vivo, and > 10 hours in E. coli in vivo. The instability index is 38.64 which classifies Fvax as stable (<40). In addition, aliphatic index of 78.08 indicates thermal stability while the negative value of its GRAVY (-0.463) classifies it to be hydrophilic. 

*Secondary structure of Fvax*

**Figure 3:**
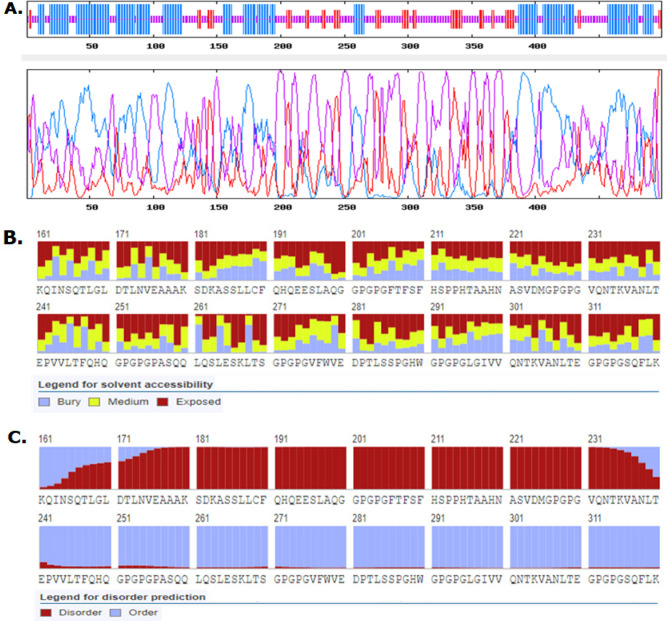
Graphical Representations of Secondary Structures, Solvent Accessibility and Disordered Regions in Fvax. Predicted secondary structures include alpha helices (39.08%) in blue, extended strands (12.63%) in red, and random coils (48.30%) in purple (A); exposed (red), medium exposed (yellow), and buried (blue) regions (B); ordered (blue) and disordered (red) regions (C)

Figure 3A shows that majority of the secondary structures in Fvax are random coils, alpha helix, and extended strands. The series of linear BL epitopes (181-250) adjoined in Fvax are random coils and extended strands. Evaluation of solvent accessibility shows that 63% of residues are exposed, 16% have medium exposure while 19% are buried; thus, majority of the residues in Fvax are exposed. Figure 3B shows that the series of linear BL epitopes (181-250) are also exposed. In line with these, 152 residues (30%) in Fvax are within the disordered regions. Favourably, the 3 candidate BL epitopes in Fvax are within the disordered regions (Figure 3C) which make these sequences more prone to the binding of BCRs in immune cells. 

*Prediction, refinement, and validation of the tertiary structure of Fvax*

**Figure 4:**
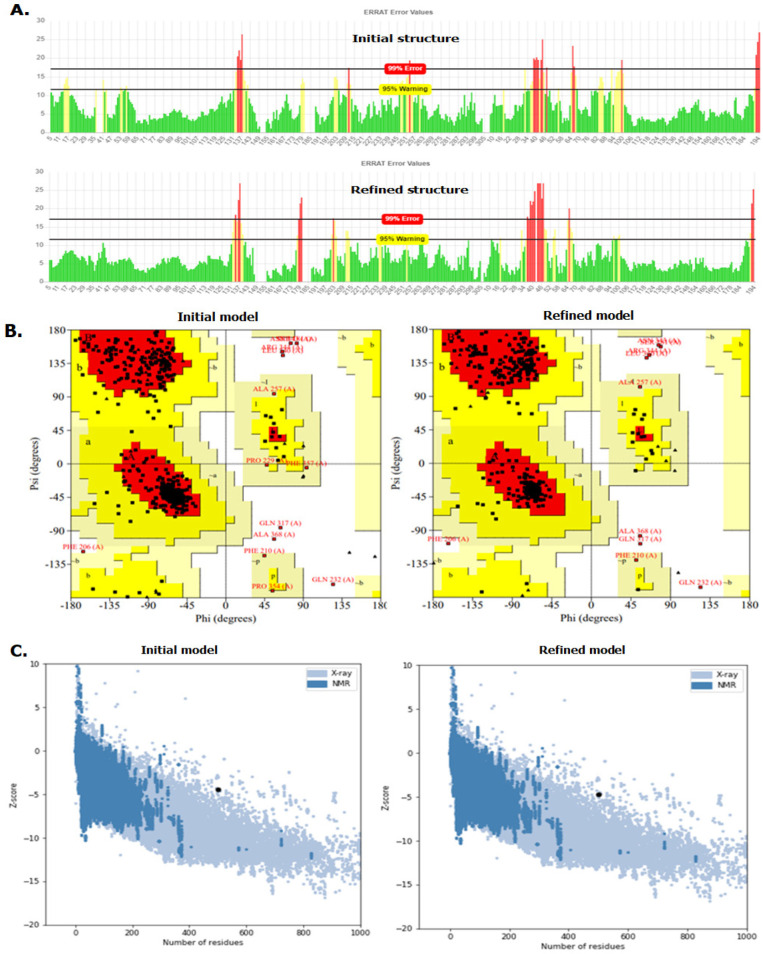
Graphical Representations of Error Values of the Residues (A), Ramachandran plots (B), and Z-score Plots (C) of Initial and Refined Model of Fvax

**Figure 5:**
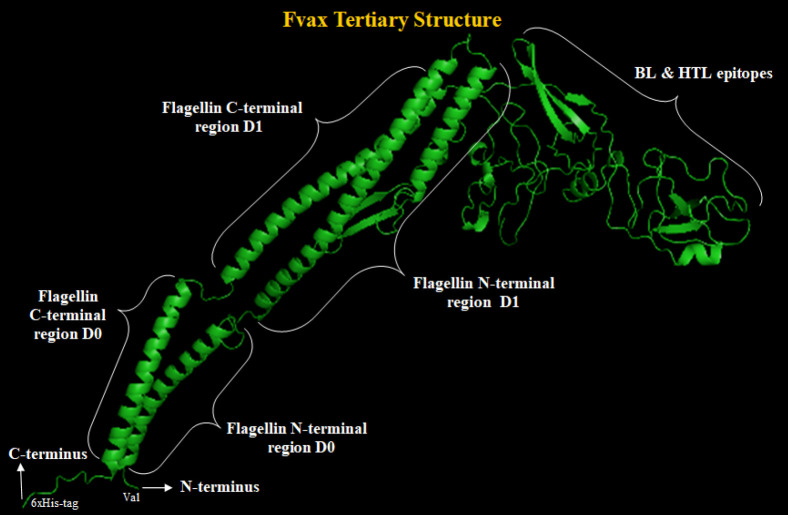
Tertiary Structure Model of Fvax Viewed in Pymol

Predicted tertiary structure models were validated and further refined for improvement. The quality of the initial structure has notably improved in the refined version. ERRAT quality factor increased from 85.13 to 89.43 (Figure 4A). Ramachandran plot values of initial model has improved from 87.9% to 92.4% for most favoured regions, 9.5% to 5.2% for additional allowed regions, 0.7% to 0.5% for generously allowed region, and 1.9% to 1.9% for disallowed regions (Figure 4B). Figure 4C shows the plot of the z-scores of the initial tertiary structure (A) and the refined version (B) which lay closer to the shaded region of the native structures (-4.47 to -4.67). Figure 5 shows the best tertiary structure model of Fvax chosen for the prediction of structural BL epitopes.

*Structural BL epitopes*

**Figure 6:**
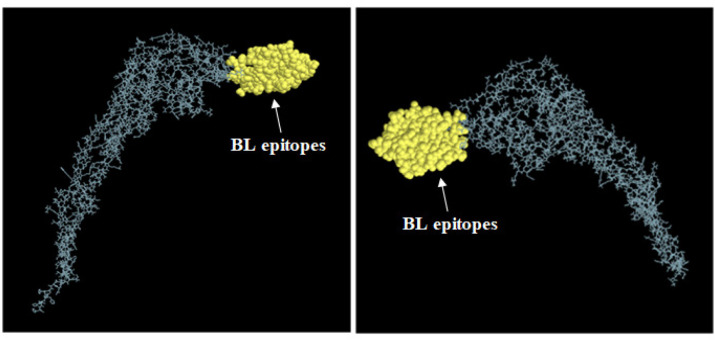
Positions of Structural Epitopes nos. 2 and 8 in the Tertiary Structure of Fvax

To further assess if the 3 linear BL epitopes included in Fvax are still accessible enough to bind with BCRs though they are adjoined with other sequences forming a tertiary structure, the protruded regions on the tertiary structure of Fvax, known here as structural BL epitopes, were identified. Ellipro predicted 7 linear (Table 3) and 3 discontinuous BL epitopes (Table 4) with protrusion index (PI) ranging from 0.553 to 0.818. Almost all of the aa residues of the 3 candidate linear BL epitopes included in Fvax (181-250) are part of both discontinuous epitope no. 8 (189-273) and linear epitope no. 2 (189-273) with high PI (0.79). These data indicate that the BL epitopes included in the vaccine (Figure 6) are highly protruded for the binding of BCRs, making Fvax more immunogenic.

*In silico cloning optimization of Fvax*

**Figure 7:**
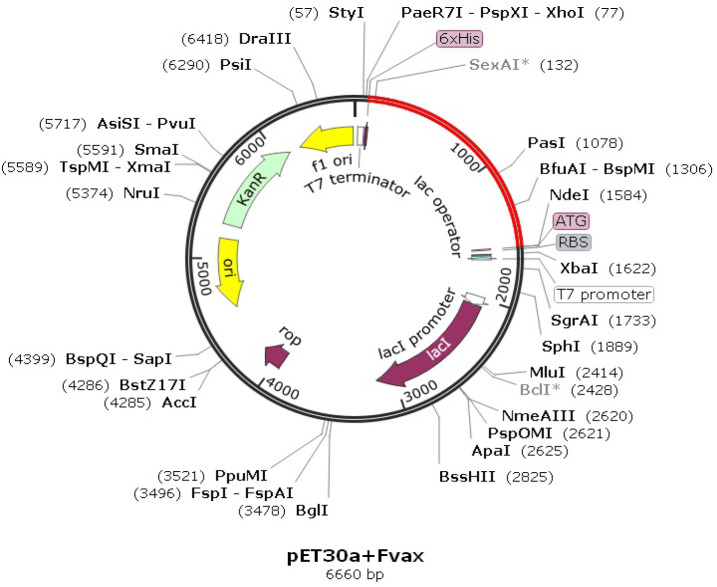
Map of Fvax Clone (6660 bp). The pET-30a (+) vector is in black (5153bp) and the optimized Fvax sequence in red (1507bp). The NdeI and XhoI restriction sites are at the N- and C-terminal of Fvax gene, respectively

The CAI-value for the optimized codons of Fvax is 0.92 which is very close to 1. While GC-content is 55.64% which is within the optimal range (30%-70%). These results convey that the optimized codons of Fvax can be highly expressed in E. coli K12 strain as host. Figure 7 shows pET-30a(+) vector (black) which contains the Fvax-encoding gene (red).

**Table 1:** Candidate BL Epitopes

Epitope	Start	End	Antigenicity score
SDKASSLLCFQHQEESLAQG	113	132	0.5978
FTFSFHSPPHTAAHNASVDM	157	176	0.5035
VQNTKVANLTEPVVLTFQHQ	317	336	0.7225

**Table 2:** Candidate HTL Epitopes

Epitope	Start	End	Antigenicity Score	IC_50_ (min-max)	MHC II Allele
PASQQLQSLESKLTS	204	218	0.7646	18 – 335	HLA-DRB1*01:01, HLA-DRB1*04:01, HLA-DRB1*04:04, HLA-DRB1*04:05, HLA-DRB1*07:01, HLA-DRB1*09:01, HLA-DRB1*11:01, HLA-DRB1*15:01, HLA-DRB4*01:01, HLA-DRB5*01:01
VFWVEDPTLSSPGHW	347	361	0.5624	128 – 400	HLA-DRB1*01:01, HLA-DRB1*03:01, HLA-DRB1*04:01, HLA-DRB3*01:01
LGIVVQNTKVANLTE	313	327	0.5526	194 – 412	HLA-DRB1*01:01, HLA-DRB1*07:01, HLA-DRB1*08:02, HLA-DRB1*13:02
SQFLKHPQKASRRPS	187	201	0.5505	113 – 454	HLA-DRB1*01:01, HLA-DRB1*11:01, HLA-DRB1*12:01, HLA-DRB5*01:01
YHFCLYWNRHAGRLH	88	102	0.6198	99 – 410	HLA-DRB1*01:01, HLA-DRB1*04:01, HLA-DRB1*04:04, HLA-DRB1*07:01, HLA-DRB1*09:01, HLA-DRB1*11:01, HLA-DRB1*15:01, HLA-DRB5*01:01
SFTFSFHSPPHTAAH	156	170	0.6298	99 – 433	HLA-DRB1*01:01, HLA-DRB1*04:01, HLA-DRB1*04:04, HLA-DRB1*07:01, HLA-DRB1*09:01, HLA-DRB1*15:01
EPVVLTFQHQLQPKN	204	218	0.6536	229 – 493	HLA-DRB1*01:01, HLA-DRB1*04:01, HLA-DRB1*04:04, HLA-DRB1*04:05, HLA-DRB1*07:01, HLA-DRB1*11:01, HLA-DRB4*01:01, HLA-DRB5*01:01

**Table 3:** Linear BL Epitopes on Fvax

No.	Start	End	Epitopes	Residues	PI
1	453	499	EDSDYATEVSNMSRAQILQQAGTSVLAQANQVPQNVLSLLRHHHHHH	47	0.818
2	189	273	CFQHQEESLAQGGPGPGFTFSFHSPPHTAAHNASVDMGPGPGVQNTKVANLTEPVVLTFQHQGPGPGPASQQLQSLESKLTSGPG	85	0.79
3	1	47	VAQVINTNSLSLLTQNNLNKSQSALGTAIERLSSGLRINSAKDDAAG	47	0.739
4	90	112	QRVRELAVQSANSTNSQSDLDSI	23	0.701
5	300	308	VQNTKVANL	9	0.664
6	400	415	ATTTENPLQKIDAALA	16	0.65
7	174	181	NVEAAAKS	8	0.553

**Table 4:** Discontinuous Epitopes on Fvax

No.	Discontinuous epitopes	Residues	PI
8	A:C189,A:F190,A:Q191,A:H192,A:Q193,A:E194,A:E195,A:S196,A:L197,A:A198,A:Q199,A:G200,A:G201,A:P202,A:G203,A:P204,A:G205,A:F206,A:T207,A:F208,A:S209,A:F210,A:H211,A:S212,A:P213,A:P214,A:H215,A:T216,A:A217,A:A218,A:H219,A:N220,A:A221,A:S222,A:V223,A:D224,A:M225,A:G226,A:P227,A:G228,A:P229,A:G230,A:V231,A:Q232,A:N233,A:T234,A:K235,A:V236,A:A237,A:N238,A:L239,A:T240,A:E241,A:P242,A:V243,A:V244,A:L245,A:T246,A:F247,A:Q248,A:H249,A:Q250,A:G251,A:P252,A:G253,A:P254,A:G255,A:P256,A:A257,A:S258,A:Q259,A:Q260,A:L261,A:Q262,A:S263,A:L264,A:E265,A:S266,A:K267,A:L268,A:T269,A:S270,A:G271,A:P272,A:G273	85	0.79
9	A:V1,A:A2,A:Q3,A:V4,A:I5,A:N6,A:T7,A:N8,A:S9,A:L10,A:S11,A:L12,A:L13,A:T14,A:Q15,A:N16,A:N17,A:L18,A:N19,A:K20,A:S21,A:Q22,A:S23,A:A24,A:L25,A:G26,A:T27,A:A28,A:I29,A:E30,A:R31,A:L32,A:S33,A:S34,A:G35,A:L36,A:R37,A:I38,A:N39,A:S40,A:A41,A:K42,A:D43,A:D44,A:A45,A:A46,A:G47,A:E453,A:D454,A:S455,A:D456,A:Y457,A:A458,A:T459,A:E460,A:V461,A:S462,A:N463,A:M464,A:S465,A:R466,A:A467,A:Q468,A:I469,A:L470,A:Q471,A:Q472,A:A473,A:G474,A:T475,A:S476,A:V477,A:L478,A:A479,A:Q480,A:A481,A:N482,A:Q483,A:V484,A:P485,A:Q486,A:N487,A:V488,A:L489,A:S490,A:L491,A:L492,A:R493,A:H494,A:H495,A:H496,A:H497,A:H498,A:H499	94	0.778
10	A:V92,A:R93,A:E94,A:L95,A:A96,A:V97,A:Q98,A:S99,A:A100,A:N101,A:S102,A:T103,A:N104,A:S105,A:Q106,A:S107,A:D108,A:L109,A:D110,A:S111,A:I112,A:E115,A:L168,A:G169,A:L173,A:N174,A:V175,A:E176,A:A177,A:A178,A:A179,A:K180,A:S181,A:V300,A:Q301,A:N302,A:T303,A:K304,A:V305,A:A306,A:N307,A:L308,A:G355,A:S356,A:F357,A:A399,A:A400,A:T401,A:T402,A:T403,A:E404,A:N405,A:P406,A:L407,A:Q408,A:K409,A:I410,A:D411,A:A412,A:A413,A:L414,A:A415,A:D418,A:T419,A:S422	65	0.626

**Figure 8:**
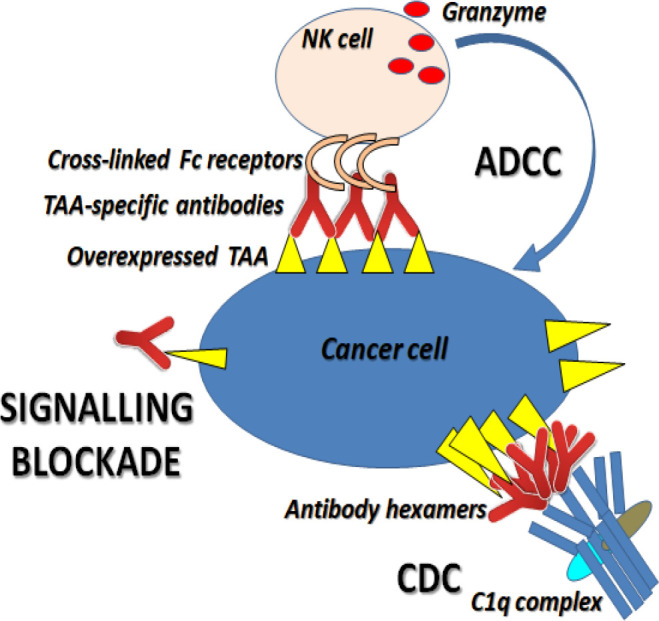
Anti-Tumor Mechanisms against Overexpressed TAA on Cancer Cells

## Discussion

The reported overexpression of GPR56 in NSCLC, which was further confirmed in this study, and its association with the mechanisms involved in the progression of NSCLC, motivated the researcher to find novel BL and HTL epitopes from the extracellular domain of GPR56. Evaluated antigenic and immunogenic sequences of GPR56 using various immunoinformatics tools can potentially activate immune responses against GPR56-overexpressing NSCLC. Studies suggest that anti-tumor mechanisms against overexpressed receptors involve signalling blockade which affects cancer-associated pathways, complement-dependent cytotoxicity (CDC) and antibody-dependent cell-mediated cytotoxicity (ADCC) (Clynes et al., 2000; Prang et al., 2005). Most of these mechanisms require cross-linking of antibodies on the surface of tumor cells or certain receptors on effector cells, making these mechanisms more effective towards cancer cells due to the overexpression of TAAs on their surface (Figure 8). On the other hand, it becomes an advantage for normal cells because they have lower expression of TAAs; thus, there can be fewer side effects.

The activation of BL using peptide-based vaccines requires the presentation of MHCII-peptide complex to an activated HTL. One of the major challenges in the development of peptide vaccines is the MHC haplotype-restricted antigen recognition. Thus, this study included HTL epitopes with good binding affinity towards the most frequent MHC II alleles. Candidate HTL epitopes have high PC worldwide (83.81%), and more importantly, have large PC (60.8% to 90.2%) in several areas with high incidence rate of lung cancer (majority is NSCLC) emphasizing the immunogenicity of Fvax as a potential immunotherapeutic agent.

Due to the intrinsic nature of peptides from GPR56, the presence of PAMPs in biomolecules known to bind to PRRs, is required to elicit immune response. In this study, the type of PRR activated by a specific adjuvant was carefully considered. Activation of some TLRs was reported to have tumor-stimulating activities (He et al., 2007; Chatterjee et al., 2014). On the other hand, TLR5 signalling was found to have anti-tumor effects in NSCLC cells (Zhou et al., 2014). A well studied PAMP known to activate TLR5 is flagellin. Flagellin has four domains, namely, D0, D1, D2 and D3. Studies showed that highly conserved D0 and D1 play critical roles in binding and activating TLR5 (Forstnerič et al., 2017; Song et al., 2017). The binding of flagellin with TLR5 was also demonstrated to enhance antitumor immune responses (Cai et al., 2011; Leigh et al., 2014). Due to reported efficacy, safety and anti-tumor benefits, flagellin was incorporated as an adjuvant in this work. And to avoid other sequences that may result to adverse reactions, only D1 and D0 were fused with the epitopes of Fvax.

The form and stability of vaccines should be considered to ensure efficient delivery of targeted actions. This work focused on developing multi-epitope vaccine which has several advantages over single peptides. Superiorities include simultaneous induction of immune response due to the adjoined antigenic and promiscuous epitopes, linked adjuvant which increases immunogenicity of the epitopes, and exclusion of sequences that can result to adverse effects. Fvax construct consists of 3 linear BL and 7 HTL epitopes which were assessed to be antigenic, non-allergenic and non-toxic. Epitopes were adjoined using GPGPG linkers proven to present sufficient epitopes in vivo (Jin et al., 2009). While flexible EAAAK linkers were used to preserve the bioactivity (Arai et al., 2001) of flagellin D0-D1 in Fvax. The addition of valine residue at the N-terminus of Fvax confers stability as implied by the drastic increase in its half-life from 4.4 hours (without valine) to 100 hours in mammalian reticulocytes. Moreover, the instability index classifies Fvax to be stable which implies longer protein half-life in vivo based from its dipeptide composition (Guruprasad et al., 1990). In addition, its GRAVY value indicates that Fvax is hydrophilic; thus, it can be easily dissolved in polar solvents like water.

Data from this study showed that the whole Fvax construct is antigenic using tumor as target. The antigenicity of epitopes is also dependent to their exposure in the vaccine conformation where BCRs can easily access. The secondary structures of the candidate BL epitopes (181-250) are random coils and extended strands, indicating exposure of these sequences. These epitopes are also found within the disordered regions of Fvax. Disordered regions have less folding structures; thus, sequences are less likely buried. The linear BL epitopes are also located within the solvent accessible regions which further emphasize their exposure. The tertiary structure of Fvax was predicted, refined, and validated. Results from the analysis justified the need for refinement and validated the tertiary structure model of Fvax. Prediction of structural epitopes also showed that the 3 linear BL epitopes included in the vaccine are highly protruded and accessible in the tertiary structure of Fvax. 

This work further evaluated the safety of Fvax as an immunotherapeutic agent. Vaccines that generate immune responses against very similar protein sequences in humans may result to adverse reactions. Favourably, Fvax was not found to have significant sequence similarity with other proteins expressed in humans except for GPR56, indicating its specificity towards the target. The construct was also classified as non-allergen, and non-toxic indicating its safety as a potential immunotherapeutic agent.

In conclusion, this is the first work to design a vaccine against GPR56-overexpressing NSCLC. In silico evaluations showed that Fvax confers stability, safety, and contains epitopes that can potentially stimulate BL and HTL immune responses. Nonetheless, the application of Fvax is anticipated to be authenticated both in vitro and in vivo.
